# Healthcare-Seeking Behaviour for Obstetric Complications in Ethiopia: A Multilevel Mixed-Effect Analysis

**DOI:** 10.1177/11786329251347353

**Published:** 2025-06-24

**Authors:** Alehegn Bishaw Geremew, Claire T. Roberts, Shahid Ullah, Jacqueline H. Stephens

**Affiliations:** 1 Flinders Health and Medical Research Institute, College of Medicine and Public Health, Flinders University, Adelaide, SA, Australia; 2 Institute of Public Health, College of Medicine and Health Sciences, University of Gondar, Gondar, Ethiopia

**Keywords:** obstetrics, pregnancy, complications, childbirth, healthcare seeking

## Abstract

**Background:**

Healthcare-seeking behaviour, and its associated factors, for obstetric complications are an important determinant of maternal deaths and adverse foetal outcomes. However, there is limited evidence on healthcare-seeking behaviours from health facilities in response to obstetric complications among Ethiopian women.

**Objective:**

To investigate women’s healthcare-seeking behaviour in response to obstetric complications, and its associated factors, in Ethiopia.

**Methods:**

Data were sourced from the Performance Monitoring for Action (PMA-E) longitudinal survey national database. Andersen’s health service use model was utilised to group individual and contextual factors. A multilevel mixed-effect logistic regression model was employed, with adjusted Odds Ratio (aOR) and 95% confidence intervals reported.

**Results:**

Data were derived from a weighted sample of 1750 women who experienced obstetric complications during pregnancy, childbirth, and immediately postpartum. Overall healthcare-seeking at health facilities for obstetric complication symptoms during the maternity continuum was 62% (95%CI: 59.6-64.3), with 47.8% (95%CI: 45.0-50.5), 64.5% (95%CI: 61.3-67.5), and 52% (95%CI: 48.3-55.6) seeking healthcare from health facilities during pregnancy, childbirth, and the immediate post-partum period, respectively. Antenatal care attendance (aOR = 3.43, 95%CI: 2.4-5.0), nulliparity (aOR = 2.1; 95%CI: 1.0-4.4), household access to media (aOR = 1.5, 95%CI: 1.0-2.1), no intimate partner violence (IPV) during pregnancy (aOR = 1.8, 95%CI: 1.1-3.1), high community wealth status (aOR = 1.2, 95%CI: 1.1-2.4), community encouragement of facility childbirth (aOR = 2.2, 95%CI: 1.1-4.3), community non-acceptance of the traditional birth attendant (TBA; aOR = 2.4, 95%CI: 1.6-3.7), and high community participation in health developmental army (HDA; aOR = 2.1, 95%CI: 1.1-3.9) were significantly associated with healthcare seeking behaviour.

**Conclusions:**

The healthcare-seeking behaviour of women from health facilities in response to obstetric complication symptoms was low and varied across the different stages of the maternity continuum. Key programme priority interventions should focus on reducing community reliance on TBA care, enhancing community encouragement of facility childbirth, and strengthening the HDA.

## Introduction

Worldwide, approximately 800 women die every day due to preventable complications associated with pregnancy and childbirth.^1^ This equated to an estimated 287 000 maternal deaths in 2020,^1^ with 94% of deaths occurring in low- and middle-income countries.^1,2^ Globally, an estimated 11.4 million untreated obstetric complications occur annually, and 951 million women have no access to emergency obstetric and neonatal care (EmONC) services.^3^ Furthermore, more than half of women who experienced pregnancy-related complications did not receive EmONC services, with large inequality existing in the met needs of EmONC in high-income countries (99%) compared to low-income countries (21%).^3^ Women's failure to get timely treatment for obstetric complications increases the risk of adverse pregnancy-related outcomes, including mortality and adverse foetal outcomes.^4,5^ According to the World Health Organisation (WHO), 15% of pregnant women develop life-threatening complications and need emergency obstetric care services.^6^ A systematic review and meta-analysis reported 18% of Ethiopian women develop potential life-threatening pregnancy-related complications.^7^ In Ethiopia, 43.1% of women experienced at least 1 pregnancy-related complication,^8^ with a maternal near miss ratio of 92 per 1000 live births,^9^ and more than 10,000 maternal deaths annually.^1^ These are mainly due to obstetric haemorrhage, hypertensive disorder of pregnancy, uterine rupture, sepsis, obstructed labour, and miscarriage.^10,11^ The provision of emergency obstetric care services provides the opportunity to reduce the severity of pregnancy-related morbidity and interrupt the chain of outcomes that potentially result in maternal and newborn mortality.^5^ Women’s timely health care-seeking behaviour for pregnancy complications, accessibility, and quality of EmONC services are crucial in reducing both severe maternal morbidity and mortality.^5,12^

Healthcare-seeking behaviour is defined as any actions individuals take when they believe they have health issues or are feeling unwell, with the intention of finding a remedy or solution.^13^ This is a proactive response to illness or health-related problems to control complications.^14^ When women experience signs and symptoms of complications during the pregnancy continuum, taking action may include seeking care from health professionals/health facilities or relying on traditional or alternative remedies. Evidence suggests a significant number of women who experience an obstetric complication in low and middle-income countries initially seek care from informal providers, such as traditional birth attendants, mothers-in-law, or elderly women in the community.^15-18^ However, the World Health Organization recommends women with obstetric complications receive medical care from those authorised to provide formal health services,^19^ as this has been shown to reduce severe maternal morbidity, mortality, and adverse foetal outcomes.

Numerous initiatives have been implemented relating to maternal and child health in Ethiopia to improve access and utilisation of maternal and child health services,^20-24^ This include policy to ensure user-free maternal healthcare at public health facilities,^25^ the Health Extension Worker (HEW) programme to provide universal access to primary healthcare,^26^ ambulance services targeted for obstetric emergency,^27^ strengthening emergency obstetric care services, Health Developmental Army (HDA) to increase the efficiency of HEW to reach every household,^22^ and maternity waiting homes aimed to facilitate access to care for high-risk pregnant women and geographically difficult to access health facility.^28^ These initiatives ultimately contribute to reducing maternal morbidity and mortality.^29-31^ However, Ethiopia is still 1 of 4 nations worldwide with a high number of maternal death,^1^ and one of six countries where half of the world’s stillbirths occur.^32^ These deaths are linked with poor healthcare-seeking behaviour by women for pregnancy complications.^12,33^

In Ethiopia, some of the challenges of improving access to, and use of, maternal health services are women’s low autonomy in health care decision-making,^34,35^ cultural norms and preferences for TBA care,^35,36^ access to emergency transportation (eg, availability and lack of response from ambulance drivers),^37^ and insufficient monitoring of community-based interventions (eg, inadequate monitoring and supervision of HDA activities).^38^ These, and other challenges, influence deciding to seek care and reaching obstetric health facilities when experiencing obstetric emergency. Delays to deciding to seek care and reaching health facility remains a major challenge in accessing maternal health services and contributes to more than 70% of maternal deaths.^39^

A multitude of factors, including age,^40^ residency,^8^ educational status,^40^ household wealth status,^40,41^ antenatal care (ANC),^41^ childbirth place,^42^ health extension worker advice,^16^ cell phone use,^16^ participation in the health developmental army,^30,43^ availability of transport,^40^ distance to health facility,^8,40^ types of complications,^40^ and availability of an emergency obstetric facility,^40^ have been found to affect healthcare-seeking behaviour for obstetric complications. Social factors, including community norms and culturally accepted practices, also influence care seeking from health facilities for obstetric complications.^18^ For example, cultural practices can confine women to bed during the postpartum period and restrict seeking health care when women develop complications during this critical period.^15,16^

Evidence is scarce about the factors affecting healthcare-seeking practice for obstetric complications, particularly across the pregnancy continuum: during pregnancy, childbirth, and postpartum. This is despite care-seeking practice across this pregnancy complication continuum being a predictor of time-specific maternal mortality.^44^ Healthcare-seeking behaviour for obstetric complications could be different across the pregnancy continuum; for example, some symptoms of complications during pregnancy are not considered illness, and in such conditions, women fail or delay seeking healthcare from health facilities.^17,40^ The determinants of healthcare-seeking behaviour for complications may exist beyond individual factors. However, there is a lack of evidence on individual and contextual factors in healthcare-seeking behaviour for obstetric complications across the pregnancy continuum.

In this study, we use Andersen and Davidson’s health behavioural model for health services^45,46^ to understand individual and contextual factors affecting healthcare seeking for obstetric complications across the pregnancy continuum. This model classifies factors affecting health services into 2 categories. The *contextual factors* are measured at some cumulative level: community characteristics, provider, and organisation/programme-related factors.^46^ The *individual factors* are measured at the women’s/household level. A multilevel mixed-effects analysis can provide valid statistical estimates for both individual and contextual factors for health service use within a hierarchical data structure.^47,48^ The individual and contextual factors affecting healthcare seeking for obstetric complications are important in implementing context-specific interventions to increase access to care for maternity continuum complications.

Herein, this study provides clear epidemiological data on healthcare-seeking behaviour for obstetric complications across the maternity continuum (pregnancy, childbirth and immediate postpartum) using population-based nationally representative data which examined individual and contextual factors, offering crucial evidence for the implementation of maternal health services in Ethiopia. The study’s objective was to investigate healthcare-seeking behaviour for obstetric complications and associated factors across the maternity continuum among Ethiopian women. The findings add to the literature on healthcare-seeking behaviour explicitly for complications during pregnancy, childbirth and immediate postpartum period and examine key context-level factors, such as community norms, community TBA acceptance, community wealth status, community access to mass media, and HDA participation. Additionally, we have used Andersen’s health behavioural model for health services and examined both individual and contextual factors crucial for impactful maternal healthcare interventions. The findings will help policymakers and programmers to improve the healthcare-seeking behaviour of women with obstetric complications.

## Methods

### Study Design

A retrospective cross-sectional study sourced data from a national (PMA-E) longitudinal survey dataset of pregnant and/or postpartum women. We present our findings following the STROBE guidelines for reporting observational studies (see Supplemental Material 1).^49^

### Setting

Ethiopia is the second most populated country in Africa with an estimated population of 129 million people, of which 50.2% are female.^50^ The country is divided into 11 regional states and two city administrations. However, the data was collected when there were only 9 regions. This study used data collected from 6 regions, including: Tigray, Afar, Amhara, Oromia, Southern Nations, Nationalities, and Peoples’ Region (SNNP), and Addis Ababa. These 6 regions account for approximately 90% of the population living in Ethiopia.^51^ The Performance Monitoring for Action (PMA-E) longitudinal survey baseline data were collected from October to December 2019 and a follow-up survey was conducted between October 2019 and September 2020.

### Data Sources

Data were requested and extracted from the PMA website.^52^ The baseline and 6-week postpartum follow-up data of the PMA-E longitudinal survey was used for this analysis. The PMA-E longitudinal survey was conducted by a collaboration of Addis Ababa University (AAU), Johns Hopkins Bloomberg School of Public Health (JHSPH), and the Federal Ministry of Health (FMOH) Ethiopia and used a 2-stage stratified cluster sampling procedure to select the participants. Women who were pregnant or postpartum were screened for the PMA-E longitudinal study, then a baseline interview was conducted. For participants who were 5 to 6 weeks postpartum at enrolment, the baseline and 6-week follow-up data were collected simultaneously. During enrolment, only baseline data from pregnant and early postpartum (0-4 weeks) participants were collected, and the 6-week follow-up interview was conducted 5 to 9 weeks after their childbirth. The detailed questionnaire development, data collection methods, and sampling procedure are available elsewhere.^51^ However, briefly, the PMA-Ethiopia questionnaire was piloted in the Oromia zone and Addis Ababa, and used in PMA Ethiopia’s 6-week postpartum maternal and newborn technical report.^53^ The region where pilot-testing occurred accounts for 33% (two-sixths) of the total region covered by the PMA-Ethiopia study. The questionnaire is available on the Johns Hopkins’ research data repository: https://doi.org/10.34976/h75w-8084.^54^

### Study Population

The study population consisted of pregnant and/or postpartum women who completed the PMA Ethiopia baseline survey. In the PMA-E longitudinal survey, 2889 women were screened, and 2855 women completed the baseline interviews. Out of those who completed the baseline survey, 2665 women completed 6-week follow-up surveys, while 190 women were not interviewed at 6 weeks postpartum follow-up due to different reasons: 92 had moved and were lost to follow-up, 54 were not at home, 20 women’s forms were missing, 15 refused to participate, 2 died, and 7 were not interviewed for other reasons; these participants were excluded from this analysis.

The study sample was women with at least 1 obstetric complication symptom during pregnancy, childbirth or the immediate postpartum period. In our study, only women with at least 1 obstetric complication symptom during the pregnancy continuum (during pregnancy and/or childbirth and/or immediate postpartum) were extracted from the PMA-E 6-week follow-up survey data. WHO suggested the symptoms of major causes of obstetric complications can be effectively illustrated through interviews with women.^19^ For this, the study population was women who had reported symptoms of obstetric complications. The following complications were reported: vaginal bleeding, high fever, convulsion/fits, severe headache with blurred vision, lower abdominal pain, high blood pressure, oedema, and worsening vision.^19,55^ Women were considered to have childbirth complications if they reported one of the following complications: vaginal bleeding during labour, leaking membrane for more than 24 hours before labour pain, malpresentation (the foetal part engaging in the maternal pelvis is other than the head)/position, leaking/rupture of membrane before 9 months, prolonged labour, and convulsion/fits.^19,55^ Women were considered as having a maternal immediate postpartum complication if they reported one of the following: retained placenta >30 minutes, high fever with foul/smelly discharge/abdominal pain, excessive bleeding (postpartum haemorrhage), and convulsion. Thus, women who had pregnancy or/and childbirth, or/and immediate postpartum complications were included in this analysis.

Women who had a termination of pregnancy (spontaneous or induced) were excluded from our analyses of healthcare seeking for childbirth complications and immediate postpartum complications. However, in the analyses of healthcare seeking for pregnancy complications and overall obstetric complications, women with spontaneous termination of pregnancy were included.

### Study Variables

#### Outcome Variables

The outcome variable was healthcare-seeking behaviour for symptoms of obstetric complications from a health facility. Women were interviewed explicitly about healthcare-seeking behaviour for obstetric complications symptoms across the pregnancy continuum: during pregnancy, childbirth, and immediate postpartum complications. Healthcare-seeking behaviour for symptoms of obstetric complications was binary yes/no, with “yes” if healthcare was sought from a health facility at least once while experiencing complications during pregnancy or childbirth or postpartum) otherwise, “no.”

The timing of obstetric complications and death can be measured across the 3 time windows: during pregnancy, childbirth, and postpartum.^44^ Thus, we did a stratified analysis of healthcare-seeking behaviour for obstetric complications symptoms during pregnancy, childbirth, or immediate postpartum. These include: (I) healthcare-seeking behaviour from the health facility for *pregnancy* complications symptoms; (II) healthcare-seeking behaviour from the health facility for *childbirth* complications symptoms; and (III) healthcare-seeking behaviour from the health facility for *immediate postpartum* (<24 hours) complications symptoms. Each outcome was coded binary yes/no, with “yes” if healthcare was sought from a health facility otherwise, “no.” In this study, health facilities include public health facilities, private health facilities, and non-governmental health facilities.

#### Independent Variables

According to the reviewed literature and theoretical knowledge, the independent variables included in our analysis were: age,^40^ marital status,^56^ educational status,^37,40,56^ family size, wealth status,^40,41^ access to cell phone, access to media, intimate partner violence (IPV),^57^ parity, ANC use,^40,41^ maternity waiting home use, pregnancy intention, Health Developmental Army (HDA) participation,^43^ health extension worker (HEW) visit,^37^ residency,^8,56^ community norms,^16,18,37^ community wealth status, community literacy, community access to media, community HDA participation, community HEW visits, and maternity home use. Access to media was measured from household access to television and radio. These variables were merged and categorised as “yes” when the household responded yes for either of the 2 and “no” when the response was no for both variables. The mean or median was used to generate context-level variables from individual data based on the data distribution. The descriptions and categories of some key variables included in the study are described in Supplemental Material 2.

### Theoretical Framework

**Figure 1.**
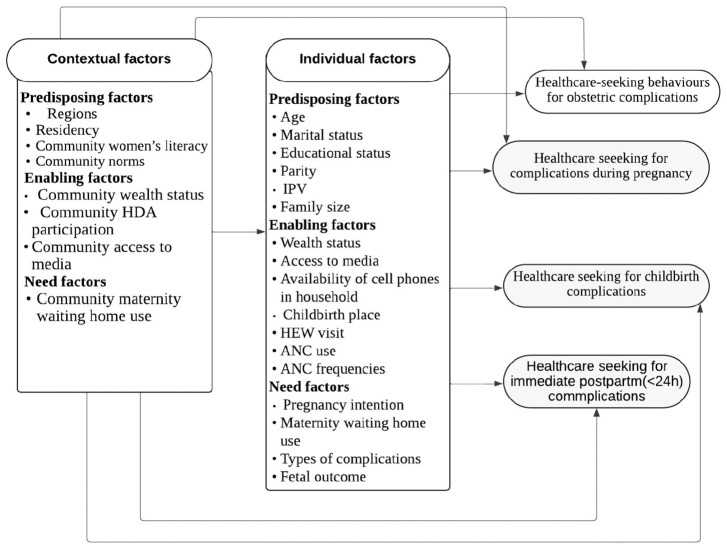
The adapted Andersen’s health services use framework shows the hypothetical factors for healthcare-seeking behaviour for obstetric complications during pregnancy, childbirth, and immediate postpartum.

Andersen and Davidson’s behavioural health model for health services use^45,46^ was adapted to select important factors of healthcare-seeking behaviours for obstetric complications and illustrate the relationship between variables. This model categorises factors into individual and contextual factors. *Individual-level variables* are measured solely based on personal/household characteristics. *Contextual-level variables* can be either direct or indirect variables and were generated from the individual-level variables in the community. Those contextual and individual-level factors were further grouped as predisposing factors: the demographic and social factors; enabling factors, which are economic (wealth status) and other facilitating factors; and need factors related to the illness symptoms. Contextual variables can influence healthcare-seeking behaviour in 2 ways: through individual characteristics and/or directly influencing healthcare-seeking behaviour, as shown in Figure 1.

### Statistical Analyses

Data management and statistical analyses were performed using Stata version 18 (StataCorp LP, College Station, TX, USA). The data were weighted to adjust for the participants’ unequal probability of selection and non-response. Participants who responded “do not know” for some of the variables were included, while other missingness was managed using single mean imputation and a complete case analysis. Normality was assessed by visual inspection of histograms and confirmed with a formal Shapiro-Wilk test. A Q-Q plot was also generated to inspect the distribution of the residuals for normality. The mean and standard deviation were used for normally distributed data, while the median and interquartile range were used for skewed data.

A multilevel (2-level) mixed-effect logistic regression model was employed to identify contextual and individual-level factors. Four models were employed for each outcome variable.^47,48^ First, the null model with only random intercept without predictors was fitted to identify the extent of cluster variability on the outcome variables. The enumeration area (clusters) was used as a random variable to estimate healthcare-seeking behaviour variation between clusters. The random effects for enumeration areas are included to account for the fact that women within the same areas may be more similar to each other than to women in others. The main effects (fixed effect) refer to the factors that are being examined in relation to the outcomes.

Random effects were assessed using log-likelihood ratio, intra-class correlation coefficient (ICC), median odds ratio (MOR), and proportion change in variance (PCV). These all measure the random effect. ICC is the proportion of variation explained by clusters. MOR to quantify the heterogeneity between groups by comparing odds across different clusters. When selected randomly, MOR is the average value of the odds ratio between high and low healthcare-seeking behaviour clusters; it shows the variance on a meaningful scale.^48,58^ PCV is the total variation of the outcome explained by the final model. Multicollinearity was checked using Variable Inflation Factors (VIF) separately for individual and context-level variables, with VIF > 5 for each variable considered to have multicollinearity.^59^

Models I (individual-level variables), II (context-level variables), and III (Individual and context-level variables) were fitted. The model comparison was made using a log-likelihood ratio (LLR) and deviance value (−2LLR), and the model with the lowest deviance value was considered the best model.^48,58,60^ Variables with *P*-value < .25 from the bivariate model, and other variables with known clinical/practical significance supported by the literature, were entered into the multivariable analysis. Adjusted odds ratios with 95% confidence intervals were used to report a measure of association (fixed effect) between independent variables with each outcome variable.

## Results

### Descriptive Characteristics of Participants

Table 1 presents the total 1750 participants who met the inclusion criteria and were included in this analysis. The breakdown of participants by regions is as follows: 776 (44.4%) from Oromia, 390 (22.3%) from Amhara, 371 (21.1%) from SNNP, 120 (6.8%) from Tigray, 65 (3.7%) from Addis Ababa, and 28 (1.6%) from Afar. Of all participants, 1296 women reported complications during pregnancy, with less women reporting childbirth complications (n = 938) and immediate postpartum complications (n = 750). The average age of the participants was 27.49 years (SD: 6.2) and most were married (n = 1660, 94.9%). More than three-quarters (n = 1375, 78.5%) of the participants were rural dwellers, and a large proportion (n = 737, 42.1%) were never educated. Nearly two-thirds of participants’ households had access to media, and most (n = 1219, 70%) had access to a cell phone. Slightly more than half (n = 923, 52.7%) of the participants were from low-literate communities. Approximately 1 in every 5 women (n = 315, 18%) were primipara. Approximately two-thirds (n = 1139, 65.1%) of participants’ pregnancies were intended. One in every 5 participants (n = 338, 19.3%) reported experiencing intimate partner violence (IPV) during pregnancy. Nearly 3 of every 4 women had at least 1 ANC use during the index pregnancy.

**Table 1.** Descriptive statistics of participants’ socio-demographic and maternal health services use with healthcare-seeking behaviour for obstetric complications in Ethiopia (N = 1750).

Characteristics	Categories	Participants distribution		Overall healthcare seeking for obstetric complications (n = 1750)		Healthcare seeking for pregnancy complications (n = 1296)		Healthcare seeking for childbirth complications (n = 938)		Healthcare seeking for immediate postpartum complications (n = 750)	
		Frequency	%	Frequency	%	Frequency	%	Frequency	%	Frequency	%
Age	<20	194	11.1	117	60.3	68	47.6	57	60.5	28	48.1
	20-24	402	22.9	269	69.9	135	47.9	183	72.4	104	59.2
	25-29	521	22.9	332	63.7	190	49.5	178	66.8	112	51.1
	30-34	334	19.1	204	61.1	122	49	103	57.5	80	50.5
	35+	299	17.1	162	54.2	103	43.3	84	57.7	71	47.4
Residency	Urban	375	21.4	277	73.9	129	48.3	178	93.2	103	79.8
	Rural	1375	78.6	808	58.7	490	47.6	426	57.0	287	46.2
Religions	Orthodox	711	40.6	477	67.1	260	48.7	287	68.8	187	57.8
	Protestant	434	24.8	238	54.8	119	39.4	139	55.7	87	43.6
	Muslim	575	32.9	355	61.7	234	53.4	167	65.0	111	50.6
	Others	30	1.7	16	53.3	6	28.5	11	78.5	5	71.4
Marital status	Married	1660	94.9	1026	61.8	591	48.0	562	63.9	366	51.5
	Others*	90	5.1	60	65.7	28	43.6	43	72.6	24	61.4
Education status	uneducated	737	42.1	408	55.4	276	47.7	191	53.8	138	42.8
	Primary school	715	40.8	447	62.5	240	46.3	258	62.8	166	52.3
	Secondary school +	298	17.1	230	77.1	103	51.5	156	90.5	86	77.1
Wealth status	Poorest	358	20.5	175	49	113	41.2	85	44.4	52	31.7
	Poorer	349	19.9	187	53.6	121	45.6	96	50.3	60	37.9
	Middle	341	19.5	209	61.4	114	45.4	114	62.3	76	52.2
	Richer	367	21	254	69.2	156	57.4	128	71.1	96	63.2
	Richest	334	19.1	258	77.2	114	48.9	182	94.0	108	81.4
Family size	⩽3	545	31.2	380	69.7	192	50.2	238	76.0	131	63.2
	3-5	597	34.1	366	61.3	211	47.8	189	61.0	138	50.7
	>5	608	34.7	338	55.7	217	46.8	177	56.3	122	44,9
Household cell phone access	No	531	30.3	285	53.7	178	45	148	51.4	94	37.7
	Yes	1219	69.7	780	65.6	440	48.9	457	70.3	295	59.2
Access to media	No	1117	63.8	641	55.4	376	45.4	348	58.0	230	44.6
	Yes	633	36.2	444	70.1	243	52.1	257	75.8	160	62.9
Community women’s literacy	Low	923	52.7	539	58.4	310	49	235	75.3	149	41.9
	High	827	47.3	546	66	309	47.1	370	55.6	241	60.2
Community health status	Low	1073	61.3	599	55.8	360	44.4	303	52.6	190	40.0
	High	677	38.7	485	71.7	259	53.3	302	83.2	200	72.7
Community access to media	Low	1157	61.1	686	59.3	428	49.5	352	56.2	250	47.4
	High	593	33.9	399	67.3	191	44.3	253	81.1	140	62.9
Parity	Nullipara	315	18	231	73.4	113	50.7	163	83.4	75	70.1
	Para 1-4	1033	59.1	634	61.4	357	47.3	340	62.5	237	50.8
	Para (⩾5)	402	22.9	219	54.5	149	46.8	102	51.4	78	44.3
IPV	No	1412	80.7	873	61.9	493	47.5	488	67.8	307	54.1
	Yes	338	19.3	212	62.7	126	48.4	117	53.2	83	45.5
ANC	No	421	24.1	158	37.5	86	27.3	77	37.7	62	32.9
	Yes	1329	75.9	927	69.7	533	54.2	527	71.9	328	58.3
HDA participation	No	1451	82.9	868	59.8	390	45.6	485	62.9	296	49.0
	Yes	299	17.1	217	72.6	129	58.1	119	71.6	95	65.0
Community HDA participation	Low	924	52.8	511	53.3	301	44.8	270	55.4	165	39.4
	High	825	47.2	574	69.5	318	50.9	335	74.1	245	64.3
Pregnancy intentions	Unintended	611	34.9	340	55.6	198	43.7	189	57.6	124	48.9
	Intended	1139	65.1	74	65.4	421	49.9	416	62.2	266	53.5
Community encourages ANC	No people	207	11.8	92	44.6	53	36.9	38	34.4	35	33.2
	Few people	290	16.6	152	52.5	101	43.4	78	50.2	48	43.1
	Some people	384	21.9	248	64.6	136	46.9	140	66.3	82	49.3
	Most people	848	48.5	579	68.3	319	52.1	343	76.2	220	61.3
	I do not know	21	1.2	12	57.1	10	60.6	6	54.5	5	62.5
Community encourages facility delivery	No people	228	13.1	82	35.8	60	34.8	27	26.0	22	23.4
	Few people	291	16.6	160	55	96	43	86	50.3	52	43.3
	Some people	338	19.3	222	65.7	127	49.4	125	63.7	86	50.5
	Most people	871	49.8	608	69.8	327	52.3	359	76.6	226	62.4
	I do not know	22	1.2	13	59	10	52.6	8	80.0	3	42.8
Community encourages PNC	No people	336	19.2	178	52.8	105	40.5	87	51.3	68	40.2
	Few people	342	19.5	197	57.7	114	43.4	102	53.7	76	51.3
	Some people	362	20.7	228	62.9	139	51.6	125	63.6	70	48.6
	Most people	682	38.9	462	67.8	249	51.7	282	76.0	170	60.1
	I do not know	28	1.2	19	67.9	12	52.2	9	81.8	7	63.6
Community TBA acceptability	Most people	330	18.9	147	44.6	87	35.2	75	47.1	43	33.5
	Some people	257	14.7	140	54.5	80	39.9	70	54.6	46	43.4
	Few people	446	25.7	273	61.2	155	47.4	158	61.0	100	49.7
	No people	690	39.4	508	73.6	287	57.4	294	76.9	197	64.6
	I do not know	27	1.5	15	55.5	10	53.5	8	80.0	5	55.5
MWH use(n = 1687)	No	1404	83.3	-		-		415	56.1	291	46.0
	Yes	283	16.7	-		-		190	95.6	99	84.5
Community MWH use	Low	994	59.0	-		-		270	53.1	190	43.5
	High	693	41.0	-		-		335	77.9	196	64.5
Place of delivery (n = 1687)	Home	745	44.2			-		-		51	14.0
	Health facility	942	55.8					-		339	88.4
Foetal outcome (n = 1687)	Livebirths	1656	98.2	-		-		-		380	51.7
	Stillbirths	31	1.8	-		-		-		10	62.5
HEW visited within 2 d after childbirth	No	1655	98.2	-		-				377	51.4
	Yes	32	1.8	-		-		-		13	76.4

* -never married, widowed, divorced.

Abbreviations: HDA, health developmental army; HEW, health extension worker; MWH-maternity waiting home.

### Obstetric Complications

**Figure 2.**
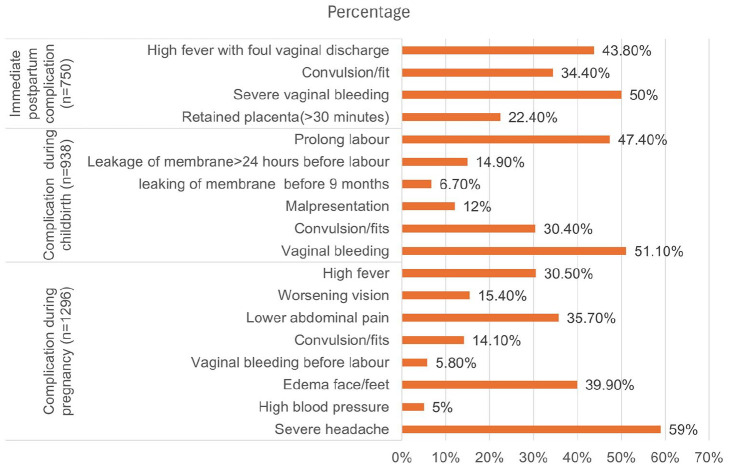
Proportion of self-reported obstetric complications among women who had obstetric complications in Ethiopia.

Of women who had self-reported complications during pregnancy, 765 (59%) had severe headaches. Among women who had childbirth complications, slightly more than half (n = 480, 51.2%) of women had severe bleeding during childbirth; similarly, from those who reported immediate postpartum complications, 1 in every 2 women (n = 375, 50%) had severe vaginal bleeding after childbirth (Figure 2).

### Healthcare-Seeking Behaviour for Obstetric Complications

**Figure 3.**
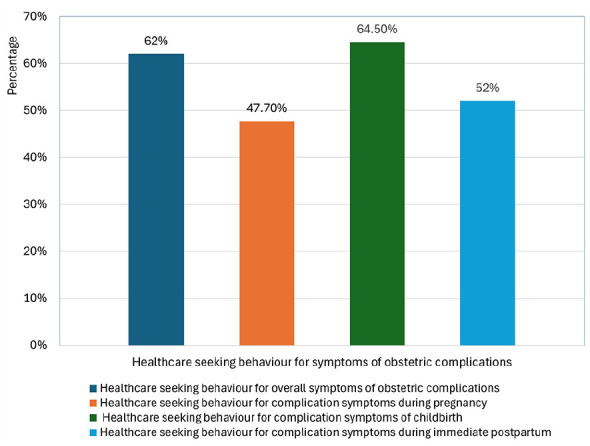
Healthcare-seeking behaviour for obstetric complications among postpartum women in Ethiopia.

The overall healthcare-seeking behaviour for obstetric complications from health facilities was 62% (95%CI: 59.6-64.3; Figure 3). This suggests much effort is needed to achieve national maternal health targets, particularly to meet the need for emergency obstetric and newborn health care services. Table 1 includes the descriptive findings of healthcare-seeking behaviours. The healthcare-seeking behaviour for symptoms of pregnancy complications was 47.7% (95%CI: 45.0-50.5). Healthcare was sought by 53 (76.8%) women with high blood pressure, 47 (62.9%) women with vaginal bleeding before labour, 343 (44.8%) women with severe headache, 63 (34.5%) women with convulsion or fits, 164 (41.1%) women with high fever, 168 (36.1%) women with lower abdominal pain, 145 (32.9%) women with oedema of face or feet, and 41 (20.0%) women with worsening of their vision during pregnancy.

Among those who had symptoms of childbirth complications, 64.5% (95%CI: 61.3-67.5) had healthcare-seeking behaviour. Healthcare was sought by 164 (57.5%) women who experienced convulsion or fits, 315 (61.8%) women who had bleeding before delivery, 76 (67.3%) women with malpresentation/foetal positional issues, 95 (67.9%) women who reported leakage of the membrane more than 24 hours before labour pain, 44 (69.8%) women who reported leaking/rupture of membrane before 9 months, and 329 (74.1%) women who experienced prolonged labour.

Among participants with immediate postpartum complications symptoms, 52% (95%CI: 48.3-55.6) had sought healthcare from a health facility. Healthcare was sought by 74 (44.0%) women who had retained placenta, 151 (45.9%) women who experienced high fever with foul discharge/abdominal pain, 235 (62.7%) women who had severe vaginal bleeding, and 141 (54.6%) women who had convulsion or fits.

### Determinants of Healthcare-Seeking Behaviour for Obstetric Complications

Community wealth status, ANC use, community facility childbirth encouragement, and community TBA acceptability were found to be statistically associated with healthcare seeking for overall obstetric complications (Table 2). The odds of healthcare-seeking behaviour for obstetric complications among women from high-community wealth status was 1.24 times (aOR = 1.24, 95%CI: 1.07-2.24) higher than those from low community wealth status. The odds of healthcare-seeking behaviour among women with ANC use was 3 times higher (aOR = 3.43, 95%CI: 2.35-5.02) than those without ANC use. The odds of healthcare seeking were 2 times (aOR = 2.17, 95%CI: 1.10-4.15) higher amongst women living in communities where birthing in a healthcare facility was encouraged by most people compared with where it was not encouraged in the community. Women from a community where TBA were not acceptable had 2 times (aOR = 2.43, 95%CI: 1.61-3.65) more odds of healthcare-seeking behaviour compared with women from a community where TBA were acceptable.

**Table 2.** Multivariable multilevel logistic regression analysis of healthcare-seeking behaviour for symptoms of obstetric complications in Ethiopia.

Variables	Null model	Model I	Model II	Model II
		AOR (95%CI)	AOR (95%CI)	AOR (95%CI)
*Age*				
<20		0.84 (0.50,1.40)		0.98 (0.60,1.61)
20-24		1.20 (0.78,1.84)		1.20 (0.80,1.80)
25-29		1		1
30-34		0.95 (0.65,1.41)		0.95 (0.64,1.39)
35+		0.74 (0.46,1.21)		0.74 (0.45,1.23)
*Women education status*				
Never educated		0.91 (0.53,1.57)		0.97 (0.56,1.66)
Primary school		1.0 (0.64,1.54)		1.04 (0.68,1.61)
Secondary and above		1		1
*Household wealth status*				
Poorest		1		1
Poorer		1.11 (0.70,1.77)		1.08 (0.67,1.73)
Middle		1.18 (0.72,1.95)		1.07 (0.64,1.78)
Richer		1.73 (0.99,3.0)		1.56 (0.81,2.99)
Richest		1.93 (1.1,3.5)*		1.77 (0.75,4.17)
*Household access to cell phone*				
No		1		1
Yes		1.15 (0.75,1.77)		1.24 (0.86,1.77)
*Access to media*				
No		1		1
Yes		1.20 (0.86,1.67)		1.25 (0.81,1.94)
*Pregnancy intentions*				
Unintended		1		1
Intended		1.24 (0.91,1.69		1.29 (0.94,1.77)
*Parity*				
Nullipara		1.50 (0.95,2.36)		1.12 (0.70,1.70)
Para 1-4		1		1
Para ⩾ 5		1.2 (0.74,1.85)		1.35 (0.85,2.15)
*IPV*				
No		1		1
Yes		0.87 (0.59,1.27)		0.85 (0.58,1.24)
*ANC*				
No		1		1
Yes		3.62 (2.38,5.49)*		3.43 (2.35,5.02)*
*HDA participation*				
No		1		
Yes		1.44 (0.98,2.14)		1.24 (0.82,1.88)
*Residency*				
Urban			1	1
Rural			1.02 (0.91,3.16)	1.14 (0.56,2.32)
*Community literacy*			1	1
Low			1	
High			1.09 (0.71,1.67)	0.90 (0.56,1.44)
*Community wealth status*				
Low			1	
High			1.85 (1.11,3.10)*	1.24 (1.07,2.24)*
*Community access to media*				
Low			1	
High			1.13 (0.77,1.66)	0.89 (0.58,1.36)
*Community HDA participation*				
Low			1	1
High			1.67 (1.17,2.39)*	1.26 (0.86,1.83)
*Community encourages ANC*				
No			1	1
A few people			1.32 (0.75,2.30)	1.18 (0.68,2.04)
Some people			1.41 (0.76, 2.61)	1.27 (0.69,2.34)
Most people			1.29 (0.65,2.57)	1.10 (0.65,2.30)
I do not know			-	-
*Community encourages facility delivery*				
No			1	1
A few people			1.88 (1.01,3.49)*	1.22 (1.15,4.26) *
Some people			2.42 (2.24,4.70)*	2.64 (1.28,5.40) *
Most people			2.07 (1.06,4.02)*	2.17 (1.10,4.25) *
I do not know			-	-
*Community encourages PNC*				
No			1	1
A few people			0.9 (0.54,1.51)	0.95 (0.57, 1.60)
Some people			0.89 (0.54,1.45)	0.87 (0.52,1.46)
Most people			0.85 (0.45,1.59)	0.78 (0.40, 1.52)
I do not know			-	-
*Community TBA acceptability*				
Most people			1	1
Some people			1.12 (0.68,1.82)	1.12 (0.68,1.84)
A few people			1.36 (0.85,2.19)	1.29 (0.81,2.06)
No			2.46 (1.63,3.37)*	2.43 (1.61,3.65)*
I do not know			-	-
*Random effect*				
Cluster variance	1.17(0.23)	0.83 (0.20)	0.62 (0.17)	0.60 (0.18)
ICC%	26.2(19,34)	20.1 (14,29)	15.8 (9.8,24)	15.5 (9,25)
MOV	3.0	2.2	1.8	1.7
PCV	Reference	29%	47%	48.7%
*Model fitness*				
Log-likelihood	−1099.22	−1026.0	−1060.39	−1005.05
Deviance	2198.44	2052	2120.78	2010.10
AIC	2202.4	2092.0	2166.78	2088.11

* = significant at *P* value < .05 and 95%CI not included 1.

Abbreviations: AIC, akaike information criteria; ICC, intraclass-correlation coefficients; PCV, proportional change variance; MOR. median odds ratio; SE, standard error.

#### Determinants of Healthcare-Seeking Behaviour for Pregnancy Complications

As presented in Table 3, the following factors were significantly associated with healthcare-seeking behaviour for complications during pregnancy: household access to media, community wealth status, ANC use, household access to cell phone, and community TBA acceptability. Women with household access to media had 1.47 times (aOR = 1.47, 95%CI: 1.02-2.10) more odds of healthcare-seeking behaviour than those without media access. Women from high community wealth status were 2.09 times (aOR = 2.09, 95%CI: 1.08-4.03) more likely to have healthcare-seeking behaviour than those from low community wealth status. Women who had ANC were 3 times (aOR = 3.12, 95%CI: 2.16-4.51) more likely to have healthcare-seeking behaviour. Women who had HDA participation had 1.63 times (aOR = 1.63, 95%CI: 1.04-2.55) more odds of healthcare-seeking behaviour compared with those who had not participated. Women from the community where TBA was not accepted by people were 2 times (aOR = 2.03, 95%CI: 1.33-3.10) more likely to have healthcare-seeking behaviour compared with those who did not.

**Table 3.** Multivariable multilevel logistic regression analysis of healthcare-seeking behaviour for symptoms of pregnancy complication in Ethiopia.

Variables	Null model	Model I	Model II	Model II
		aOR (95%CI)	aOR (95%CI)	aOR (95%CI)
*Age*				
<20		0. 74 (0.43, 1.28)		0.80 (0.46,1.39)
20-24		0.91 (0.63, 1.31)		0.93 (0.64,1.34)
25-29		1		1
30-34		0.96 (0.65, 1.41)		1.01 (0.69,1.49)
35+		0.85 (0.55, 1.32)		0.85 (0.55,1.33)
*Women education status*				
Never educated		1.25 (0.79, 1.97)		1.18 (0.74,1.89)
Primary school		1.14 (0.77, 1.68)		1.13 (0.76,1.67)
Secondary and above school		1		1
*Household wealth status*				
Poorest		1		1
Poorer		1.49 (0.92,2.40)		1.42 (0.88,2.29)
Middle		1.26 (0.76, 2.08)		1.11 (0.67,1.84)
Richer		1.79 (1.05, 3.05)*		1.19 (0.6,2.23)
Richest		0.98 (0.53, 1.82)		0.6 (0.25,1.46)
*Household access to cell phone*				
No		1		1
Yes		1.19 (0.84, 1.69)		1.38 (0.98,1.94)
*Household media access*				
No		1		1
Yes		1.26 (0.91, 1.75)		1.47 (1.02,2.10)*
*Pregnancy intentions*				
Unintended		1		1
Intended		1.13 (0.85, 1.49)		1.10 (0.83,1.46)
*Parity*				
Nullipara		1		1
Para 1-4		1.25 (0.84,1.85)		1.18 (0.79,1.76)
Para ⩾5		1.18 (0.79,1.76)		1.20 (0.80,1.79)
*IPV*				
No		0.90 (0.63,1.29)		0.85 (0.59,1.21)
Yes		1		1
*ANC*				
No		1		1
Yes		3.26 (2.26, 4.69)*		3.12 (2.16,4.51)*
*HDA participation*				
No		1		1
Yes		1.10 (0.76, 1.58)		1.16 (0.79,1.70)
*Residency*				
Urban			1	1
Rural			1.52 (0.84,2.77)	1.07 (0.54,2.12)
*Community literacy*				
Low			1	
High			0.77 (0.49,1.21)	0.76 (0.47,1.25)
*Community wealth status*				
Low			1	1
High			2.19 (1.26,3.80)*	2.09 (1.08,4.03)*
*Community access to media*				
Low			1	1
High			0.78 (0.49,1.24)	0.72 (0.43,1.20)
*Community HDA participation*				
Low			1	1
High			1.12 (0.79,1.58)	0.86 (0.58,1.28)
*Community encourages ANC*				
No			1	1
A few people			1.37 (0.78,2.41)	1.26 (0.70,2.27)
Some people			1.72 (0.99,2.99)	1.51 (0.85,2.67)
Most people			1.62 (0.96,2.73)	1.31 (0.76,2.25)
I do not know			-	-
*Community TBA acceptability*				
Most people			1	1
Some people			1.14 (0.7,1.83)	1.10 (0.68,1.79)
A few people			1.22 (0.79,1.88)	1.13 (0.72,1.76)
No			2.16 (1.43,3.75)*	2.03 (1.33,3.10) *
I do not know				
*Random effects*				
Cluster variance (SE)	0.90(0.20)	0.83 (0.20)	0.64 (0.17)	0.69 (0.18)
ICC%	21.5(15,30)	20.2 (13.5,29)	16.3 (10.4,24)	17.4 (11,26)
*Model fitness*				
Deviance	1717.4	1651.6	1745	1623.3
Log-likelihood	−858.71	−825.82	−872.51	−811.68
AIC	1724.42	1691.65	1711.08	1689.37

* = significant at *P* value < .05% and 95% CI not included 1.

#### Determinants of ealthcare-Seeking for Childbirth Complications Symptoms

Age, household wealth status, parity, ANC use, IPV during pregnancy, community HDA participation, and community-encouraged facility childbirth were associated with healthcare-seeking behaviour for childbirth complications (Table 4). Women from the wealthiest households had 6 times (aOR = 6.70, 95%CI: 1.70-26.0) the odds of healthcare seeking for childbirth complications than those of the poorest household wealth status. The authors acknowledged a large aOR and a wide confidence interval for the wealthiest households. However, we noted that the direction of association remained consistent across bivariate and multilevel analyses (model 1 and model 3). Nullipara women were 2 times (aOR = 2.13, 95%CI: 1.03-4.40) more likely to have healthcare-seeking behaviour compared with women who had previously given birth. Women who had ANC had nearly 3 times (aOR = 2.62, 95%CI: 1.47-4.68) higher odds of healthcare seeking than those who did not use ANC. Compared with women who had IPV during pregnancy, women who had no IPV were nearly twice (aOR = 1.84, 95%CI: 1.08-3.14) more likely to have healthcare-seeking behaviour. Women from communities who encouraged childbirth in healthcare facilities were 6 times (aOR = 6.3, 95%CI: 2.62-15.18) more likely to seek healthcare than those from communities that did not.

**Table 4.** Multivariable multilevel logistic regression analysis of healthcare-seeking behaviour for symptoms of childbirth complications in Ethiopia.

Variables	Null model	Model I	Model II	Model III
		aOR (95%CI)	aOR (95%CI)	aOR (95%CI)
*Age*				
<20		1.01 (0.41,2.48)		0.98 (0.40,2.40)
20-24		2.23 (1.18,4.28)*		2.26 (1.19,4.32) *
25-29		1		1
30-34		0.75 (0.40,1.42)		0.67 (0.35,1.26)
35+		1.12 (0.52,2.40)		0.93 (0.43,1.93)
*Education status*				
Never educated		0.70 (0.31,1.59)		1.10 (0.47,2.54)
Primary school		0.66 (0.31,1.38)		0.92 (0.44,1.93)
Secondary and above school		1		1
*Household wealth status*				
Poorest		1		1
Poorer		2.17 (1.10,4.27)*		1.73 (0.88,3.40)
Meddle		2.13 (1.02,4.41)*		1.47 (0.71,3.01)
Richer		5.13 (2.25,11.69)		2.64 (1.06,6.57) *
Richest		31.6 (10.54,94.0)*		6.70 (1.70,26.0) *
*Household access to cell phone*				
No		1		1
Yes		0.76 (0.45,1.27)		0.97 (0.51,1.84)
*Access to media*				
No		1		1
Yes		0.61 (0.35,1.62)		0.63 (0.36,1.12)
*Pregnancy intentions*				
Unintended		1		1
Intended		1.23 (0.77,1.96)		1.12 (0.63,1.93)
*Parity*				
Nullipara		2.16 (1.06,4.38)		2.13 (1.03,4.40)*
Para 1-4		1		1
Para ⩾5		1.27 (0.64,2.44)		1.21 (0.64,2.30)
*IPV*				
No		1.89 (1.10.3.24)*		1.84 (1.08,3.14)*
Yes		1		1
*ANC*				
No		1		1
Yes		3.12 (1.74,5.60)*		2.62 (1.47,4.68)*
*HDA participation*				
No		1		1
Yes		1.09 (0.58,2.03)		0.87 (0.46,1.66)
*Residency*				
Urban			1	1
Rural			0.47 (0.16,1.38)	0.71(0.19-2.56)
*Community literacy*				
Low			1	1
High			1.82 (0.92,3.60)	1.58 (0.73,3.43)
*Community health status*				
Low			1	
High			2.87 (1.17,7.04)*	1.81 (0.68,4.76)
*Community access to media*				
Low			1	1
High			1.77 (0.72,4.34)	1.89 (0.81,4.39)
*Community HDA participation*				
Low			1	
High			1.66 (0.77,3.59)	1.82 (0.93,3.55)
*Community encourages health facility delivery*				
No			1	
A few people			4.43 (1.90,10.29)	5.09 (2.03,12.7)*
Some people			6.11 (2.55,14.6)*	6.78 (2.63,17.4)*
Most people			6.22 (2.77,13.96)	6.30 (2.62,15.1) *
I do not know			-	-
*Community TBA acceptability*				
Most people			1	1
Some people			1.34 (0.66,2.72)	1.58 (0.73,3.39)
A few people			1.20 (0.63,2.28)	1.23 (0.62,2.45)
No			2.72 (1.41,5.24)*	2.78 (1.38,5.60)*
I do not know			-	-
*Random effect*				
Cluster variance	5.6(1.3)	2.70 (0.7))	1.51 (0.5)	1.75 (0.56)
ICC%	63(51,73)	45 (32,58)	31 (20.46)	34.7 (21.7,50)
*Model fitness*				
Log-likelihood	−453.06	−396.30	−398.64	−372.3
Deviance	906.1	792.6	798.6	744.6
AIC	910.12	832.60	827.28	808.73

* = significant *P* value < .05 and 95%CI not included 1.

#### Determinants of Healthcare-Seeking Immediate Postpartum Complications

Four variables were associated with healthcare-seeking behaviour for immediate postpartum complications (Table 5). Women who stayed in a maternity waiting home were 5 times (aOR = 5.08, 95%CI: 2.56-10.07) more likely to seek healthcare for immediate postpartum complications than those who did not. Women from communities with high participation in HDA were 2 times (aOR = 2.10, 95%CI: 1.11-3.87) more likely to seek healthcare than low HDA participation communities. Participants from communities that encouraged healthcare facility childbirth had 3 times more odds (aOR = 3.27, 95%CI: 1.16-9.20) of healthcare-seeking behaviour compared to those in the community who did not encourage facility childbirth. Finally, women from communities who did not accept TBA were 2 times (aOR = 2.46, 95%CI: 1.23-4.92) more likely to have healthcare seeking behaviour.

**Table 5.** Multivariable multilevel logistic regression analysis of healthcare-seeking behaviour for immediate postpartum (<24 hours) complications in Ethiopia.

Variables	Null model	Model I	Model II	Model III
		aOR (95%CI)	aOR (95%CI)	aOR (95%CI)
*Age*				
<20		0.79 (0.30, 2.07)		0.77 (0.27,2.03)
20-24		1.25 (0.69,2.26)		1.30 (0.71,2.36)
25-29		1		1
30-34		0.99 (0.54,1.80)		0.96 (0.52, 1.76)
35+		1.0 (0.49,2.02)		0.93 (0.46,1.89)
*Women education status*				
Never educated		0.90 (0.43,1.88)		1.14 (0.52,2.52)
Primary school		1.14 (0.58,2.24)		1.29 (0.64,2.57)
Secondary and above school		1		1
*Household wealth status*				
Poorest		1		1
Poorer		1.54 (0.79,3.02)		1.22 (0.62,2.41)
Meddle		1.92 (0.94,3.94)		1.48 (0.72,3.06)
Richer		3.99 (1.82,8.76)		2.37 (0.99,5.76)
Richest		9.26 (3.56,24.04)		3.54 (0.97,12.0)
*Household access to cell phone*				
No		1		1
Yes		1.28 (0.76,2.15)		1.33 (0.79,2.24)
*Access to media*				
No		1		1
Yes		0.87 (0.51,1.47)		0.91 (0.52,1.60)
*Pregnancy intentions*				
Unintended		1		1
Intended		0.84 (0.53,1.31)		0.86 (0.54,1.35)
*Parity*				
Nullipara		1.53 (0.75,3.12)		1.34 (0.64,2.78)
Para 1-4		1		1
Para ⩾5		1.59 (0.84,2.99)		1.52 (0.80,2.87)
*Intimate Partner Violence (IPV)*				
No		1		1
Yes		0.77 (0.46,1.28)		0.75 (0.45,1.26)
*ANC*				
No		1		
Yes		1.87 (1.06,3.30)		1.56 (0.87,2.79)
*HDA participation*				
No		1		1
Yes		1.28 (0.73,2.25)		0.91 (0.50,1.67)
*Maternity waiting home use*				
No		1		1
Yes		5.72 (2.95,11.07)		5.08 (2.56,10.0)*
*Foetal outcome*				
Live births		1		1
Stillbirths		3.51 (0.78,15.76)		3.59 (0.81,15.91)
*Residency*				
Urban			1	
Rural			0.46 (0.19,1.11)	0.58 (0.19,1.71)
*Community literacy*				
Low			1	
High			1.76 (0.93,3.29)	1.42 (0.67,2.99)
*Community health status*				
Low			1	1
High			1.93 (0.88,4.23)	1.2 (0.47,0.07)
*Community access to media*				
Low			1	1
High			1.06 (0.54,2.09)	0.87 (0.39, 1.92)
*Community HDA participation*				
Low			1	1
High			2.55 (1.51,4.28)*	2.10 (1.11,3.87)*
*Community maternity waiting home use*				
No			1	1
Yes			1.49 (0.88,2.55)	0.93 (51,1.70)
*Community encourages facility delivery*				
No			1	1
A few people			2.41 (0.96,6.04)	2.287 (0.84,6.15)
Some people			3.78 (1.48,9.62)*	3.99 (1.44,11.0)*
Most people			3.38 (1.31,8.72)*	3.27 (1.16,9.20)*
I do not know			1	1
*Community encourages PNC*				
No			1	1
A few people			1.27 (0.61,2.66)	1.31 (0.59,2.90)
Some people			0.93 (0.42,2.04)	1.02 (0.43,2.41)
Most people			1.15 (0.52,2.52)	1.15 (0.49,2.67)
I do not know			-	-
*Community TBA acceptability*				
Most people			1	
Some people			1.38 (0.66,2.87)	1.45 (0.65,3.20)
A few people			1.28 (0.66,2.46)	1.19 (0.58,2.46)
No			2.56 (1.36,4.82)	2.46 (1.23, 4.9)*
I do not know			-	-
*Random effect*				
Cluster variance (SE)	3.4(0.87)	1.6 (0.5)	0.97 (0.39)	1.2 (0.5)
ICC%	51.4(39,63)	33 (20,48)	22.7 (12,39)	26.8 (14,45)
*Model fitness*				
Deviance	955.9	781.9	796.5	744.9
Log-likelihood	−447.95	−390.99	−398.29	−372.45
AIC	899.90	825.99	837.73	824.90

* = significant at *P* value < .05 and 95%CI not included 1.

### Model Comparison and Random Effect

For all 4 outcomes, the final models had the highest log-likelihood ratio and the lowest deviance, thus, were the best-fit models. In the null model of overall healthcare-seeking behaviour for any obstetric complications, ICC was 26.2%, which indicates over a quarter of this healthcare-seeking behaviour was due to clustering, with the remaining 73.8% due to individual differences of the women. The test of log-likelihood was statistically significant (*P* < .001). In the stratified analysis, the ICC for the null model of healthcare-seeking behaviour for complications during pregnancy was 21.5%. That is, 21.5% of healthcare-seeking behaviour for pregnancy complications was due to clustering. Likewise, for childbirth and immediate postpartum complications, the ICC was 63% and 51.4%, respectively. This indicates 37% of healthcare seeking for childbirth complications and 48.6% of immediate postpartum complications were due to individual differences among the participants. Furthermore, the MOR was 3.0 for the null models of overall healthcare-seeking behaviour for overall obstetric complications. Therefore, if we randomly select women from 2 different clusters, women from clusters with high healthcare-seeking behaviour were 3 times more likely to seek healthcare for their complications compared with women from clusters with low healthcare-seeking behaviour.

## Discussion

Maternal and newborn mortality and severe morbidities are deeply intertwined with healthcare-seeking behaviour for pregnancy-related complications. This study aimed to investigate healthcare-seeking behaviour for obstetric complications and identify contextual and individual factors using Andersen’s health services use model. The findings reveal that 62% of women had healthcare-seeking behaviour for obstetric complications throughout the pregnancy continuum. The stratified analysis further explains women’s healthcare-seeking behaviour: 48% sought healthcare for complications during pregnancy, 65% sought healthcare for childbirth complications, and 52% sought healthcare for immediate postpartum complications. This finding is significantly lower than the National Reproductive Health Strategy (2021-2025) target of meeting 100% of the need for emergency obstetric and newborn care services.^61^ This implies more effort is required to improve maternal health in Ethiopia to significantly progress towards the national goal of reducing maternal mortality.

Our multivariable multilevel mixed-effect analysis identified several key factors associated with healthcare seeking for complications during the obstetric continuum of care. These include community wealth status, ANC use, community encouragement to use childbirth facilities, and community TBA acceptability. The stratified analysis further identified household access to media, community wealth status, ANC use, and community TBA acceptability were significant factors for healthcare seeking for complications during pregnancy. Similarly, for healthcare-seeking behaviour for complications during childbirth, household wealth status, parity, ANC use, IPV during pregnancy, community TBA acceptability, and community-encouraged facility childbirth were significantly associated factors. Lastly, women’s maternity waiting home use, community participation in HDA, community TBA acceptability, and community-encouraged facility childbirth were factors associated with healthcare-seeking behaviour for immediate postpartum complications.

Our findings on healthcare-seeking behaviour for obstetric complications are consistent with previous studies. Our results surpassed the prevalence reported in prior studies conducted in the Amhara and Oromia regions of Ethiopia, where 45%^16^ and 53%^37^ of women sought healthcare respectively, and in Tanzania^62^ where 34.5% of women sought early medical care when they experienced complication symptoms. An explanation for this may be that Ethiopia has been strengthening community maternal health interventions and quality improvement initiatives, as reflected by increasing maternal health service indicators for ANC, facility delivery, and postnatal care.^21^ These health service improvements may have also influenced healthcare-seeking behaviour for obstetric complications. In addition, the previous Ethiopian studies may have underestimated healthcare seeking behaviours because participants were women who had given childbirth 6 to 12 months before the interview.^16^ Therefore, the data collection could have been impacted by recall bias, with maternal complications and care-seeking practices under-reported.^55^ The inconsistency with the Tanzanian study might be the difference in measurement related to the timing of seeking care; the former measures the urgency of healthcare seeking. In contrast, our finding is lower than studies from northwest Ethiopia^8^ and Ghana^56^ where 73.8% and 73.6% of women received care from a health professional for complications, respectively. The likely reasons for the difference are that more than half of the complication cases were malaria-related in the Ethiopian study, and in the Ghana study the participants were women who had experienced miscarriage. This could have affected health-seeking behaviour, as healthcare-seeking behaviour differs by type of complications,^40^ because the perception of complication seriousness and norms related to specific complication can differ.^16^

The reasons for discrepancies in obstetric complication healthcare seeking across settings includes variation in communities’ trust of TBA care, cultural norms such as postnatal restriction with perceived spiritual and physical vulnerability,^18,36,37,63^ and differences in women’s empowerment for health decision making.^64,65^ Additionally, perceiving illness causation as supernatural or medical can influence care seeking. When women perceive illness causation as supernatural, they seek care from informal sources.^40^ Deciding to seek care and attend a healthcare facility is influenced by community sentiment, including the type and quality of local health interventions and the variety of community-based maternal interventions, which can significantly influence healthcare seeking for complications.^21,30,43^ Accessibility of emergency transportation,^37^ and inequalities in utilisation of antenatal care,^37,66^ could create differences in healthcare seeking for obstetric complications. As such, there is a need for tailored interventions and community involvement in maternal health.

Healthcare-seeking behaviour varies for complications across the maternity continuum: during pregnancy, childbirth, and immediate postpartum. This might be explained by differences in the risk perceptions of women and the community related to the timing of complications. Being unable to recognise the severity of some complications, different traditional options offered by TBA, and having the opinion care from formal providers is unnecessary for some of the complications might also be possible reasons.^8,41^ For instance, women with some pregnancy complication symptoms failed to seek healthcare from health facilities. Also, women with heavy bleeding after delivery have been show to perceive this as cleaning out of the womb and accepting it as expected.^67^ Evidence of the potential impact of not seeking care is provided by a study from Bangladesh, which showed significant proportions of women who died due to postpartum haemorrhage had not sought healthcare from health facilities.^42^ Traditionally, the restriction of women’s movement outside the home during the postpartum period, traditional care options, and a lack of awareness to seek care after childbirth may also affect healthcare seeking for postpartum complications.^16,37^ WHO recommends that women receive the first PNC within 24 hours after home delivery to detect and manage complications for mothers and newborns.^68^

Women from households in the wealthiest quantile were more positively associated with seeking healthcare for childbirth complications than those with the lowest household wealth status. Previous studies support our finding,^8,16,40^ which showed that household wealth status increases healthcare-seeking practice. Even if there is a user fee exemption policy for maternal health services at public health facilities in Ethiopia,^23^ seeking maternal healthcare for obstetric complications incurs out-of-pocket costs, such as transport and medicine costs.^69^ Moreover, a review of studies from low-income countries showed that the availability of money for transport affects healthcare-seeking practice for maternal and newborn illness.^15^ Thus, women with better household incomes could invest in transport, diagnosis, and treatment for complications.^69^ In fact, healthcare for illnesses during pregnancy and childbirth poses a substantial financial risk.^70^ Further research may be needed on household wealth inequality and the healthcare-seeking behaviour for obstetric complications.

We showed that nulliparous women were nearly 3 times more likely to have healthcare-seeking behaviour for childbirth complications compared with women who had previously given birth. We propose nulliparous women may be more cautious and have a greater fear of a poor foetal outcome, thus being more motivated to seek complication-related healthcare than their counterparts.

Mass media is used to seek information about healthcare and social support.^71^ In our study, we showed women with household access to media have a higher likelihood of healthcare-seeking behaviour for complications during pregnancy. Studies from Afghanistan^65^ and Sub-Saharan Africa,^72^ are consistent with our findings, as were similar findings reported in Ethiopia.^72^ This implies women may use media for information seeking and disseminating information on danger signs and seeking healthcare from formal providers, enhancing healthcare-seeking practice for obstetric complications.^71^

Antenatal care increased healthcare-seeking behaviour for symptoms of obstetric complications, which has been supported by a previous study.^8^ Women receiving ANC also receive health promotion on birth preparedness and complication readiness. Thus, women are more informed about where to go when experiencing obstetric complications, thereby enabling healthcare-seeking for obstetric complications.^37^ This also improves timely recognition of danger signs.^15^ Additionally, women who have ANC may be less likely to engage in traditional options, further increasing the likelihood of healthcare-seeking for pregnancy complications. Furthermore, in the present study, we compared women who experienced IPV during pregnancy with those who had not. We found increased healthcare-seeking behaviour for childbirth complications amongst those who had not experienced IPV, which is supported by other studies.^57,73^ Most likely, IPV during pregnancy reflect low partner support and affects women’s decision-making power regarding their health.^73^

Women who used maternity waiting homes were significantly more likely to have healthcare-seeking behaviour for postpartum complications. The likely reason is that women are probably informed of the risk of complications while at maternity waiting homes, since the primary purpose of these services are to reduce geographical barriers and prevent obstetric complications among high-risk pregnant women. Research has shown MWH reduces obstetric complications ^31^; however, in Ethiopia, only half of the health facilities have MWH, and distribution varies across the regions of Ethiopia.^29^ This implies efforts to improve women’s use of maternity waiting homes and the construction of MWHs to improve healthcare-seeking behaviour for obstetric complications.

Healthcare-seeking behaviour for obstetric complications was more likely by women living in communities that encouraged facility childbirth compared to women from communities where facility childbirth was not encouraged by the community. This likely reflects cultural beliefs and traditional practices, with some communities promoting different traditional and religious practices for women with obstetric complications rather than encouraging formal health care providers. Behavioural change interventions, including distributing educational leaflets by health extension workers and saving money for emergencies, have been suggested to improve healthcare seeking for obstetric complications.^63^ In addition, community TBA acceptance negatively affected healthcare-seeking behaviour. More specifically, compared with communities where most people accept TBA care, women in communities that did not accept TBA care were more likely to have healthcare-seeking behaviour for obstetric complications. TBA adherence to traditional practices, women’s cultural shyness, and the need for high privacy could influence women’s preference for TBA care for childbirth complications.^36^ In many communities, TBA often serve as the first point of contact for pregnant women with complications.^37^ Additionally, some TBA use herbal remedies to treat complications, such as nausea, vomiting, and labour pain, and to facilitate women’s push during labour and the management of postpartum haemorrhage.^74^ Research has shown women and their community perceive the quality of TBA care affects healthcare seeking from health facilities for obstetric complications.^64^ In addition, TBAs are involved in decision-making to seek care,^37^ which could promote their services in preference to formal healthcare providers. Other factors influencing seeking healthcare from a formal provider are the community’s cultural norms, together with transportation, geographical accessibility, and disrespectful maternity health services.^36^

Another factor significantly associated with healthcare-seeking behaviour for complications during pregnancy was community HDA participation. The HDA is an initiative to avoid delays in accessing maternal, newborn, and child health services. The government established this programme in 2012 to improve maternal and child health outcomes by strengthening the link between the healthcare system and the community. Herein, we showed women from communities with high participation in HDA had increased healthcare seeking for childbirth complications. Similarly, a previous study showed HDA increased healthcare-seeking practice for neonatal danger signs^66^ and facilitated healthcare-seeking for obstetric complications.^37^ HDA is the key source of information for women in preparing for childbirth and pregnancy-related complications. Further, HDA strengthens the linkage of women in the community with health facilities via health extension workers during childbirth and complications. The presence of HDA within 2 km distance from health facility improves maternal health services indictors.^30^ This strengthens evidence that the level of implementation and density of HDA could enhance maternal health services utilisation. Women are often not empowered to decide about their healthcare. Thus, HDA participation could allow mobility and improve women’s decision-making power.^75^ However, there have been poor functionality and inequalities the HDA implementation across the regions.^38,43^

## Strengths and Limitations

We used population-based national representative longitudinal survey data collected during pregnancy and at 5 to 9 weeks postpartum. This reasonable time data collection could reduce the recall bias of self-reported symptoms of obstetric complications and healthcare-seeking behaviour. Multilevel modelling was employed to obtain reliable estimates and stratification by healthcare seeking for pregnancy, childbirth, and immediate postpartum complications. We identified both context and individual-level factors of healthcare seeking for obstetric complications. However, this study acknowledges potential limitations. Primarily, the study participants’ eligibility was based on self-reported obstetric complications, which may have indirectly biased in measuring the outcome variable. Bias from this self-reported data occurs when participants self-assess measures of obstetric complications. The main reasons bias may have been introduced into the data are misunderstanding, recall bias (forgetting the symptoms they experienced), and social desirability bias (participants wanted to look healthy).^76^ In obstetric complication symptoms, misunderstanding and forgottenness of complication symptoms are likely, however using multiple complication symptoms and interviews conducted within a reasonable time frame should have minimised bias resulting from misunderstanding and forgotten complication symptoms.^55^ For this study, we did not compute a sample size; however, all eligible participants from the population-wide PMA Ethiopia dataset were considered,^52^ providing a good estimate to answer our research questions. Furthermore, the data were collected from regions where 90% of the population lived in the country, and stratification of rural/urban dwellers during sampling was considered, but unnecessary. Hence, the findings of this study can be applicable beyond the study context.

## Conclusion

The findings indicate varying levels of healthcare-seeking behaviour for obstetric complications across Ethiopia, with the highest prevalence during childbirth complications. A low percentage of women seeking healthcare for obstetric complications in Ethiopia is concerning, given the WHO’s recommendation that all women with obstetric complications need to receive healthcare from a formal provider. Healthcare-seeking behaviour for obstetric complications during pregnancy, childbirth, and immediate postpartum, was influenced by both individual and contextual factors. Individual factors (age, ANC, parity, household media access, household access to cell phone, house wealth status, women participation in HDA, IPV during pregnancy) and contextual factors (community wealth status, community media access, community encouragement of facility childbirth, community acceptance of TBA, community HDA participation) were found to be factors affecting healthcare seeking for obstetric complication. Furthermore, we found differences in the factors influencing healthcare-seeking for complications during pregnancy, childbirth, and the immediate postpartum period. These results contribute uniquely to the literature on healthcare-seeking practice for obstetric complications. In addition, given that healthcare-seeking behaviour following obstetric complications is critical for reducing maternal mortality and adverse foetal outcomes, strategies and policies must be directed towards the identified factors.

## Implications for Practice

This study provides significant insights into the healthcare-seeking behaviour in response to obstetric complications. Key programme priority interventions should focus on reducing community reliance on TBA care, enhancing community encouragement of facility-based childbirth, and strengthening HDA health promotion activities. Behavioural change educational interventions targeting communities reliant on TBA and where community cultural norms discourage childbirth in health facilities would improve healthcare seeking for obstetric complications. Strengthening women’s health developmental programmes through mentoring of women’s HDA activities, ensuring active engagement of HDA members, and expanding the density of HDA workers may also improve healthcare seeking for maternal complications. The government may need to redefine and better integrate TBA roles within formal health systems. Empowering the community through culturally sensitive education on the importance of healthcare seeking from health facilities for pregnancy-related complications is also needed. Strategies for preventing IPV during pregnancy may improve women’s capacity to seek healthcare for obstetric complications. Despite user-free maternity health services offered in public facilities, household wealth affects healthcare-seeking for obstetric complications; as such, measures to improve economic status and reduce women’s indirect costs for obstetric complication care services, including transport costs, will improve healthcare-seeking behaviour for complications. Media should be leveraged to disseminate information related to obstetric complications, as it is a necessary platform to enhance healthcare-seeking behaviour for obstetric complications. Investing in these initiatives will contribute to achieving national commitments to reduce adverse pregnancy outcomes and maternal deaths.

## Implications for Research

Understanding healthcare seeking for obstetric complications across the maternity continuum provides opportunities for more tailored interventions. Further qualitative research to explore community cultural norms and traditional birth attendants’ role in healthcare-seeking behaviour for obstetric complications will provide further in-depth evidence. Future researchers need to focus on better understanding healthcare seeking for obstetric complications to explicitly address pregnancy, childbirth, and postpartum complications.

## Supplemental Material

sj-docx-1-his-10.1177_11786329251347353 – Supplemental material for Healthcare-Seeking Behaviour for Obstetric Complications in Ethiopia: A Multilevel Mixed-Effect Analysis Supplemental material, sj-docx-1-his-10.1177_11786329251347353 for Healthcare-Seeking Behaviour for Obstetric Complications in Ethiopia: A Multilevel Mixed-Effect Analysis by Alehegn Bishaw Geremew, Claire T. Roberts, Shahid Ullah and Jacqueline H. Stephens in Health Services Insights

sj-docx-2-his-10.1177_11786329251347353 – Supplemental material for Healthcare-Seeking Behaviour for Obstetric Complications in Ethiopia: A Multilevel Mixed-Effect Analysis Supplemental material, sj-docx-2-his-10.1177_11786329251347353 for Healthcare-Seeking Behaviour for Obstetric Complications in Ethiopia: A Multilevel Mixed-Effect Analysis by Alehegn Bishaw Geremew, Claire T. Roberts, Shahid Ullah and Jacqueline H. Stephens in Health Services Insights
