# Supplementary material for: Healthcare-Seeking Behaviour for Obstetric Complications in Ethiopia: A Multilevel Mixed-Effect Analysis
Source: Health Serv Insights. 2025 Jun 24;18:11786329251347353. doi: 10.1177/11786329251347353 (PMC12188065; doi:10.1177/11786329251347353)
Supplement: sj-docx-2-his-10.1177_11786329251347353 – Supplemental material for Healthcare-Seeking Behaviour for Obstetric Complications in Ethiopia: A Multilevel Mixed-Effect Analysis [file sj-docx-2-his-10.1177_11786329251347353.docx]

Supplementary Material 2: The description, category and measurement of independent variables

| **Variables** | **Categories** | **Description and/or measurement** |
| --- | --- | --- |
| Age | <20,20-24,25-29,30-34, ≥35 | Age of women in years |
| Residency | Rural, Urban | Place of residency |
| Marital status | Married, others (single, widowed, divorced | Women's marital status |
| Educational status | Never education, primary school, secondary school or above | Women's highest level of educational status |
| Community-women literacy | low, high | Measured from women's literacy that able to read/write any language and the mean value of women's literacy per enumeration area were computed then women less than the mean classified as "low" while the mean and above as "high" |
| Household wealth status | Poorest, poorer, middle, richer, richest | The household wealth index (quantile) was measured using principal component analysis based on household assets. Finally, the household wealth score was classified into five categories. |
| Household cell phone access | Yes, no | Availability of cell phones in the household. |
| Household media access | Yes, no | Household access to radio/and television. Women who responded “yes” for at least one of the two were considered “yes” while no for both household media access was labelled as “no” |
| Community wealth status | low, high | The values generated from household wealth status and the median value of household wealth status per enumeration area were computed. Then, values below the median were classified as "low," while values above the mean were classified as "high." |
| HDA participation | Yes, no | Women's membership in 1 to five networks during the index pregnancy |
| Community HDA participation | low, high | Measured from individual women's participation in HDA and the median value of women's HDA participation per enumeration area (cluster) were computed. The values below the median were classified as “low” while values above the mean were as "high". |
| Maternity waiting home use | Yes, no | Women went to maternity waiting homes before labour constructed at or near health facility |
| Community maternity waiting for home use | low, high | Measured from individual women's maternity home use before labour and the median value of women's maternity home use per enumeration area (cluster) were computed. Then values below the mean were classified as "low" while values above the mean were as "high". |
| Community access to media | low, high | Measured from individual household access to media and the mean value of household access to media per enumeration area (cluster) were computed. Then values below the mean were classified as "low" while values above the mean were as "high". |
| Community encourage ANC | No people, few people, some people, most people, do not know | Each participant was interviewed and measured people in the community encourage antenatal care |
| Community thinking delivery by TBA is acceptable | No people, few people, some people, most people, do not know | Measured people in the community thought that childbirth by TBA was acceptable |
| The community encourages facility delivery | No people, few people, some people, most people, do not know | Each participant was interviewed and measured people in the community encourage facility childbirth |
| Community encourage PNC | No people, few people, some people, most people | Measured people in the community encourage postnatal care |
| Pregnancy intention | Intended, unintended | Women at enrolment were asked at the time you became pregnant, did you want to become pregnant then, did you want to wait until later, or did you not want to have any / any more children at all? The responses "then", "later" and "not all. In this analysis, women who responded "then" were labelled as "intended" while both later and not at all were labelled as "unintended". |
| Parity | Nullipara (0), para 1-4, para ≥5 | Number of live births at enrolment |
| ANC use | Yes, no | At least one ANC contact during the index pregnancy from the health facility |
| Visited by HEW after childbirth | Yes, no | Community health extension workers visited women within two days after childbirth. |
| IPV during pregnancy | Yes, no | Women were interviewed about their experience of physical and sexual violence by intimate partners during their index pregnancy time. Those women who responded "yes" for at least one question of physical/sexual IPV during pregnancy were labelled as "yes" |
